# Editorial: Sexual dimorphism in autoimmune and immune-dysregulated diseases

**DOI:** 10.3389/fimmu.2024.1531757

**Published:** 2024-12-06

**Authors:** C. Alex Colvert, Melissa A. Cunningham

**Affiliations:** Division of Rheumatology and Immunology, Department of Medicine, Medical University of South Carolina, Charleston, SC, United States

**Keywords:** autoimmune diseases, sex differences, immune dysregulation, lupus, EAE (experimental autoimmune encephalitis), rheumatoid arthritis

In 2014, the National Institutes of Health (NIH) implemented policy that specified sex as a biological variable (SABV) be considered in all research designs involving vertebrate animal and human studies. Prior to this, scientists often incorporated only male sex into scientific research (1, 2). Although it is important that SABV be investigated in all areas of health-related research, it is particularly important that sex be considered in research related to immunity and autoimmunity since women have increased incidence of autoimmune diseases compared to men. The Research Topic “Sexual Dimorphism in Autoimmune and Immune-dysregulated Diseases” serves to help advance the understanding of how sex chromosomes and sex hormones influence autoimmune and immune-dysregulated diseases.

Autoimmune and immune-dysregulated diseases are thought to arise due to a combination of genetic and environmental factors (such as infections and pollutants) that result in overactivation of the immune system. Lahita discusses how microRNA (miRNA), genetics and epigenetics, estrogen (E2), prolactin (PRL), progesterone (P4), and androgens modulate autoimmunity in his review, “*Sex and gender influence on immunity and autoimmunity*.” Lahita ultimately concludes that the female predisposition for most autoimmune diseases remains unelucidated but is likely due to a combination of the genetics of the X chromosome, hormonal regulation of immune cells, and sex hormones affecting epigenetics.

Systemic Lupus Erythematous (SLE) is an autoimmune disease characterized by autoantibody formation and immune complex deposition in target organs. SLE predominates in women, with nine women affected for every one man, and patients living with SLE are at an increased risk for cardiovascular disease (CVD). In the article “*Cardiac and vascular complications in lupus: Is there a role for sex?”* Corker et al. discuss the potential mechanisms of sex and sex hormones in the development of CVD in SLE. With regard to sex, male patients with SLE tend to have a more severe disease state that involves renal disease compared to women; this could be due to low testosterone levels in men with SLE. They discuss a clinical study by Hill et al. that reports that estrogen supplementation in a transgender woman, was possibly associated with lupus nephritis (3). Importantly, Corker et al. discuss the role of estrogen receptors, ultimately concluding that estrogen receptor alpha plays a predominant role in the sex-dependent development of SLE, however there is a significant knowledge gap in the understanding of SABV in physiological and pathophysiological mechanisms of the development of CVD complications in SLE. This is partly due to current literature investigating mainly women with SLE and female mouse models.

The connection between B cell immune responses and hormones in SLE, rheumatoid arthritis (RA), and multiple sclerosis (MS) is explored by Santana-Sánchez et al.. RA is a debilitating autoimmune disease that is characterized by inflammation of the synovial membranes in joints as well as other tissues; MS is a chronic autoimmune disease that affects the central nervous system and is characterized by extensive demyelination, axonal loss, and neuronal degeneration. In all 3 diseases, B cells play a critical role in the pathogenesis of disease. Of note, both peptide and sex hormones are involved in the homeostasis of B cells. B cells express the PRL receptor in all maturation states, suggesting that PRL could be involved in the development of B cells. E2s can directly contribute to the pathogenesis of autoimmune diseases by altering the differentiation and function of B cells. Growth hormone is also crucial for B cell interactions and is a regulatory protein in numerous B cell processes. Testosterone (T2) is known to regulate B cell tolerance and P4 is speculated to cause a decrease in B cell maturation, although less is known. The authors acknowledge that although unclear, the myriad effects of hormones on B cell function are a potential explanation for the sexual dimorphism seen in autoimmune diseases.

EAE is the most widely accepted animal model for MS. In the article “*Effects of biological sex and pregnancy in experimental autoimmune encephalomyelitis: It’s complicated*,” McCombe et al. advocate for full disclosure of the EAE model used in research studies when exploring the effects of biological sex and pregnancy. Specifically, in their conceptual analysis, they extensively review the effects of sex on incidence and severity in actively induced EAE vs. spontaneous EAE vs. adoptive transfer EAE. The authors note that the species/strain, EAE-inducing antigen, and the differences/similarities observed between males and females all greatly impact the phenotype. They also explore cells, molecules and pathways that exhibit sex differences in EAE. Lastly, they discuss the implications of hormones and pregnancy in EAE. They conclude that among animal models, the effects of sex in EAE vary substantially.

In apparent contrast, Guillain Barre syndrome (GBS) and chronic inflammatory demyelinating polyradiculoneuropathy (CDIP) are autoimmune diseases that show predominance in males and prevalence increases with age. Characterized by demyelination and axonal pathology, GBS is usually preceded by illness, infection, trauma, or surgery. GBS and CDIP are unlike other autoimmune diseases in that there is no clear target antigen or HLA association. McCombe et al. explore possible mechanisms for the sexual dimorphism seen in GBS and CIDP. They postulate that the sex differences in GBS and CDIP could be due to males becoming more susceptible to immune dysfunction with age more so than females, which may contribute to the male predominance.

In the final article of the Research Topic, Gao et al. evaluate the association between systemic immunity-inflammatory index (SII) and sex hormones in children and adolescents. Using weighted multiple regression analyses, they found a negative correlation between SII and sex-hormone binding globulin (SHBG) in male participants (mean age 12.32 +/- 3.95 years). In female participants, a negative correlation was observed between SII and T2, SHBG, and ratio of T2 to estradiol. Using subgroup analyses and interaction tests based on diabetes status, body mass index, and puberty index, they discovered that SII did not have a significant correlation with these subgroups as opposed to females, where a consistent association was seen. These results suggest that SII may predict possible risks of sex hormone disorders, setting the groundwork required for preventative strategies against such disorders through SII surveillance.

Collectively, these articles evaluate the pervasiveness of sex and sex hormones in autoimmune and immune-dysregulated diseases. This Research Topic highlights the importance of investigating SABV, as sex and sex hormones affect expression of miRNAs, epigenetic modifications, and immune cell development, among many other biochemical, immunological, and physiological functions.
